# Constructing a DNA ladder Range for Lambda Phage by multiplex PCR

**Published:** 2010-12

**Authors:** R Gopalakrishnan, S Joseph, S Sellappa

**Affiliations:** Department of Biotechnology, School of Life Sciences, Karpagam University, Coimbatore, Tamil Nadu, India.

**Keywords:** Lambda phage DNA, DNA ladder, Multiplex PCR

## Abstract

**Background and Objectives:**

DNA ladder contains DNA fragments of different length but with known size, used to determine the size of unknown DNA molecules. Different DNA ladders are available for expected DNA length. Conserved sequences were selected for design of primers to generate DNA fragments of known specific size.

**Materials and Methods:**

In this study, we describe a method by which DNA ladder was prepared based on multiplex PCR technique. Different lengths of DNA fragments were amplified using the primers designed according to the 1216-2136 sequence extent of lambda phage DNA. Target DNA fragments were amplified using multiplex PCR and extracted.

**Results:**

The results showed an amplified lambda phage DNA at particular target sites by using 1 forward and 6 different reverse primers (for 100, 200, 400, 600, 800, 1000bp) for the successful amplification.

**Conclusion:**

This method would be more cost effective than commercial DNA molecular weight markers.

## INTRODUCTION

Determining the molecular weight (mw) or the base pair (bp) length of nucleic acids in the field of molecular biology is indispensable. Preparing a DNA ladder using common PCR is laborious. Multiplex PCR is a variant of PCR which enables simultaneous amplification of numerous targets of interest in one reaction by using more than one pair of primers (1).

The current study is intended for production of novel DNA marker through successful extension of diverse target sites of purified lambda DNA sequence by using single forward primer and six different reverse primers for complementary orientation.

## MATERIALS AND METHODS

From the lambda phage DNA sequence (Gene Bank accession no: J02459), 1216–2136 bp sequences were selected and primers were synthesized (Table 1). The lambda phage DNA was isolated (2). The purified DNA was used for PCR amplification. 100, 200, 400, 600, 800, 1000 bp fragments were amplified by multiplex PCR (3) using six reverse primer pairs in one PCR tube with the single forward primer. PCR was performed using a thermo cycler with a temperature profile of 35cycles, which contained 2 cycles at 94°C for 30 s, 58°C for 30 s, and 72°C for 1 min, 2 cycles 94°C for 30 s, 54°C for 30 s and 72°C for 1min, 2 cycles for every annealing temperature interval 2 temperature, until 60°C polishing 35 cycles. PCR products were detected by 1% agarose gel at 100 V for 30 minutes in a 15 ml gel. The length was estimated by comparing the known size DNA marker (Helini biomolecules), and was then purified and sequenced. The PCR products were extracted through phenol/chloroformand precipitated with ethanol, and then analyzed their UV absorbance under 260 nm. Five µl aliquot of the material was electrophoresed and no change in the band migration pattern or band intensities was observed following ethidium bromide staining. The marker was then frozen at −20°C.

**Table 1:** Primers for DNA ladder construction.

Base pair	Sequence	Usage	Position
1000bp	3′-CATACACAATGGTCGGGTCA-5′	Reverse primer	2117–2136
800bp	3′-GCATTTCGTAGCGGTCCAG -5′	Reverse primer	1920–1938
600bp	3′-TCCAGTCTTTGACAATCTGCAC-5′	Reverse primer	1719–1740
400bp	3’-GCTCGCAGAGATAAAACACG -5’	Reverse primer	1517–1536
200bp	3′-GATGGACTTTGGCCAGACC-5′	Reverse primer	1322–1340
100bp	3′-GCCACATCCACCGACTTTT -5′	Reverse primer	1216–1234
1000bp	5′-TAACACGCTCACCATGAAGC -3′	Forward primer	1136–1155

## RESULTS

**Fig. 1:**
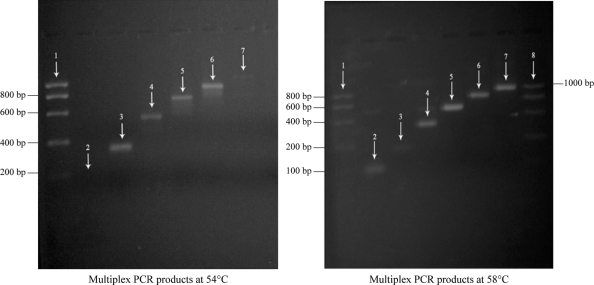
showing multiplex PCR products.

The isolated DNA was checked using UV absorbance at 260/280 nm. For successful amplification, the most desired annealing temperature for target sites in lambda phage was found to be above 56°C because 100 bp region was not amplified at 54°C. We account that the annealing temperature at 58°C was efficient for amplification of target sites (Fig. 1).

## DISCUSSION

Current study was planned for the successful amplification of different target sites by using multiplex PCR because the single suitable forward primer will move in 5′–3′ direction and different reverse primer move in complementary orientation on a purified lambda DNA.

All fragments of 100–1,000 bp were amplified successfully with multiplex PCR by optimizing the PCR reaction system and temperature profile. PCR is a frequently used technique for different study purposes (4, 5) and PCR derived techniques such as multiplex PCR, has been widely used at present to detect and identify some unknown and possible pathogenic microorganisms in some diseases could reduce costs and raise production in laboratory.
